# Application of Carbon–Silicon Hybrid Fillers Derived from Carbonised Rice Production Waste in Industrial Tread Rubber Compounds

**DOI:** 10.3390/polym17152070

**Published:** 2025-07-29

**Authors:** Valeryia V. Bobrova, Sergey V. Nechipurenko, Bayana B. Yermukhambetova, Andrei V. Kasperovich, Sergey A. Yefremov, Aigerim K. Kaiaidarova, Danelya N. Makhayeva, Galiya S. Irmukhametova, Gulzhakhan Zh. Yeligbayeva, Grigoriy A. Mun

**Affiliations:** 1 Department of Polymer Composite Materials, Faculty of Technology of Organic Substances, Belarusian State Technological University, 220006 Minsk, Belarus; bobrova@belstu.by (V.V.B.); andkasp@belstu.by (A.V.K.); 2 Faculty of Chemistry and Chemical Technology, Al-Farabi Kazakh National University, Almaty 050040, Kazakhstan; baya_yerm@mail.ru (B.B.Y.); efremsa@mail.ru (S.A.Y.); aigerim_ko@list.ru (A.K.K.); danelya.1993@gmail.com (D.N.M.); galiya.irm@gmail.com (G.S.I.); 3 National Engineering Academy of the Republic of Kazakhstan, Khodzhanova Str., 67, Almaty 050060, Kazakhstan; 4 Institute of Organic Synthesis and Carbon Chemistry of the Republic of Kazakhstan, Alikhanov Str., 1, Karaganda 100000, Kazakhstan; g.yeligbayeva@gmail.com

**Keywords:** silica filler, carbon–silicon filler, rice husk, elastomeric compounds, tread rubber, elastic hysteresis properties, elasticity, plastic deformation, tackiness

## Abstract

The disposal of agro-industrial waste is a pressing environmental issue. At the same time, due to the high silica content in specific agricultural residues, their processed products can be utilised in various industrial sectors as substitutes for commercial materials. This study investigates the key technological, physico-mechanical, and viscoelastic properties of industrial elastomeric compounds based on synthetic styrene–butadiene rubber, intended for the tread of summer passenger car tyres, when replacing the commercially used highly reinforcing silica filler (SF), Extrasil 150VD brand (white carbon black), with a carbon–silica filler (CSF). The CSF is produced by carbonising a finely ground mixture of rice production waste (rice husks and stems) in a pyrolysis furnace at 550–600 °C without oxygen. It was found that replacing 20 wt.pts. of silica filler with CSF in industrial tread formulations improves processing parameters (Mooney viscosity increases by up to 5.3%, optimal vulcanisation time by up to 9.2%), resistance to plastic deformation (by up to 7.7%), and tackiness of the rubber compounds (by 31.3–34.4%). Viscoelastic properties also improved: the loss modulus and mechanical loss tangent decreased by up to 24.0% and 14.3%, respectively; the rebound elasticity increased by up to 6.3% and fatigue resistance by up to 2.7 thousand cycles; and the internal temperature of samples decreased by 7 °C. However, a decrease in tensile strength (by 10.7–27.0%) and an increase in wear rate (up to 43.3% before and up to 22.5% after thermal ageing) were observed. Nevertheless, the overall results of this study indicate that the CSF derived from the carbonisation of rice production waste—containing both silica and carbon components—can effectively be used as a partial replacement for the commercially utilised reinforcing silica filler in the production of tread rubber for summer passenger car tyres.

## 1. Introduction

Automotive tyres with enhanced performance characteristics—such as rolling resistance, safety, durability, and environmental friendliness—must meet the continuously increasing demands of consumers. In this regard, reinforcing materials of various types, such as carbon black and colloidal silica, are added to rubber compounds to improve key properties such as modulus, hardness, tensile strength, and tear resistance. Moreover, there is a growing need to enhance and optimise critical performance indicators of tyre treads, including wet grip, rolling resistance, and wear resistance. Tyres typically contain 30–35% of various grades of carbon black, which serve as reinforcing fillers for different components of the tyre [1,2].

Since 1992, when Michelin first introduced silica (SiO_2_) as a functional filler in tread compounds for passenger car tyres [3], demonstrating that SiO_2_ additives can reduce rolling resistance and improve wet traction, silica-reinforced tyre treads have become the subject of extensive research aimed at developing tyres with enhanced performance, improved energy efficiency, and greater driving safety [4,5,6]. Agricultural crop residues—particularly rice, a staple crop central to food security and energy needs for nearly half the global population—are considered promising raw materials for producing silicon-containing fillers [7,8]. However, the annual volume of agricultural waste in the form of rice husks and straw currently reaches approximately 700 million tons, posing a serious environmental challenge, especially in countries like India. Due to their high silica content, rice husks and straw are highly resistant to biodegradation in soil and require substantial land for disposal.

Farmers often resort to open-field burning without economically viable alternatives for managing this waste. For instance, in India, an estimated 178 million tons of plant residues are burned annually, of which approximately 87 million tons consist of rice straw and husks [7,8].

It should be noted that the open burning of rice husks and straw is accompanied by the release of large volumes of harmful gases—such as methane, nitrous oxide, carbon dioxide, sulphur dioxide, volatile organic compounds, and carcinogenic polycyclic aromatic hydrocarbons—as well as aerosol particles (including coarse dust and delicate particulate matter), all of which contribute significantly to air pollution [7,8].

At the same time, the products of rational processing of rice husks and straw, which contain both silica and carbon components, possess a range of properties valuable for practical applications. However, this potential remains largely underutilised [9,10,11,12,13,14,15,16,17,18,19].

One of the promising approaches to the practical utilisation of rice husk and straw processing products is using their carbonised forms as fillers in elastomeric compositions to replace industrial fillers [20,21,22,23,24,25]. Such carbonisates are typically produced by carbonising a finely ground mixture of rice husk and stem in a pyrolysis furnace in the absence of oxygen. Due to its low cost and high silica content compared to other natural materials, rice husk and stalk have been recognised as suitable for a wide range of industrial and applied processes.

The unique physical and chemical characteristics of rice husk, namely the high content of amorphous silica (57–67 wt.% SiO_2_), developed porous structure, low density, and large specific surface area, determine its demand in various industrial processes and formulations, including as part of elastomer compositions [26,27,28]. Thus, in study [29], the physico-mechanical properties of materials based on natural rubber filled with rice husk ash (RHA) at varying loadings from 0 to 40 parts per 100 parts of rubber were investigated and compared with commercial reinforcing fillers (silica and carbon black). The results showed that incorporating RHA into natural rubber improved hardness but reduced tensile strength and tear resistance. Other properties, such as Young’s modulus and abrasion loss, showed no significant changes. However, natural rubber filled with RHA exhibited better resilience compared to rubber containing commercial silica and carbon black. Numerous attempts have also been made to utilise RHA as a filler for synthetic rubbers. In one of such attempts [30], it was found that the introduction of low-dispersity RHA into nitrile rubber-based compositions up to 60 parts by weight contributes to an insignificant change in the mechanical properties of rubbers, an improvement in the thermal stability of vulcanisates, which makes it possible to use RHA as a filler, providing both economic and environmental benefits. However, in work [31], during the study of the influence of the dosage of rice husk ash on the properties of materials based on natural rubber, it was found that the use of this filler is accompanied by a deterioration in mechanical properties (tensile strength, elastic modulus, hardness, abrasion resistance, and tear strength) compared to reinforcing fillers such as silicon dioxide and carbon black.

It is worth noting that a significant number of studies have focused on obtaining amorphous silica from RHA [32,33,34,35,36,37], which is characterised by higher reactivity and lower impurity content compared to crystalline forms of silica such as quartz and cristobalite. It has been reported that silica derived from rice husk waste through chemical treatment and subsequent calcination at 600 °C, when used at low loadings (up to 20 wt.pts.) in tread formulations based on natural rubber, enhances tensile strength, hardness, and elastic modulus while slightly decreasing elongation at break and abrasion resistance [38,39].

In the mid-1990s, Continental became one of the first companies in the tyre industry to recognise the potential of using silica (white carbon black) as a component in rubber compounds. Experimental results were promising, showing that silica exhibits its beneficial properties during mixing, where it reacts with silane to promote bonding between components. Today, tyre manufacturers continue to work on making white carbon black and other fillers more environmentally friendly. White silica is used in the tread of tyres to reduce rolling resistance, enhance wear resistance, and improve wet traction. It is also used in the breaker layer (a layered structure at the upper part of the tyre that reinforces and stabilises the tread) to reduce heat build-up, thereby contributing to reduced fuel consumption and increased environmental performance.

The use of rice husk ash in elastomeric blends for both tread and breaker layers of tyres generally follows two approaches: either exploring the partial replacement of conventional silica with rice husk-derived silica or developing formulations with special coupling agents or surface modifiers to improve the compatibility of ash with rubber [40,41]. However, both approaches typically lead to an increase in the overall production cost.

The present study aims to utilise a carbonisation product derived from agricultural waste (rice husk and stems), containing both carbon and silica fractions and not subjected to additional treatment, as a partial replacement for a commercial, highly reinforcing silica filler (white carbon black) in industrial elastomeric compounds based on styrene–butadiene rubber (SBR). This study also seeks to evaluate the technological, physico-mechanical, viscoelastic, and performance properties of the resulting materials. The goal is to reduce production costs, minimise environmental pollution from agricultural waste disposal, and impart specific properties to rubber compounds.

For this purpose, existing industrial formulations intended for the tread section of summer passenger car tyres were used, incorporating the rice husk and stem ash (carbonisate) as a substitute for the white silica grade Ultrasil 150GR.

## 2. Materials and Methods

### 2.1. Materials

Synthetic SBR was employed to prepare industrial elastomeric compounds to produce the tread part of summer car tyres. This rubber is intended to manufacture all-season and summer tyres, characterised by low rolling resistance, improved wet traction, and enhanced wear resistance (country of origin: Russian Federation).

The basic formulation of the studied rubber compound is presented in Table 1.

The main fillers in the studied rubber compound are carbon black grade N339 and a reinforcing SF, which consists of colourless silicon dioxide crystals with a melting point of +1713 to +1728 °C and is characterised by high hardness and strength (Table 2).

It is well known that incorporating SF into rubber compounds provides several advantages, such as improved tear resistance, reduced heat generation, enhanced adhesion, lower rolling resistance, and improved hysteresis behaviour in multicomponent products such as tyres [42]. The reinforcement mechanism of polymers by silica fillers differs significantly from that of carbon black due to the distinct chemical nature and surface energy characteristics of silica, which contrast sharply with those of carbon black [43,44].

As a partial replacement for the commercial reinforcing silica filler, a CSF was used, which was produced by NeoCarbon LLP. (Almaty, Kazakhstan) using a technology developed by the authors of this article [45]. The raw material consisted of rice husks (RH) and rice straw (RS) ground in a rotary knife mill to a particle size of less than 5.0 mm. The components were mixed in RS: RH ratios ranging from 2.0:5.0 to 0.5:2.0. The resulting blend was subjected to carbonisation in a pyrolysis furnace at 550–600 °C without oxygen. The obtained carbonisate was further milled to a particle size of less than 25.0 µm. The physicochemical properties of the resulting CSF are presented in Table 3 [45].

Table 4 presents the elemental composition of the CSF surface, determined using an EDX Quantox-200 fluorescence spectrometer (Bruker, Bremen, Germany).

**Figure 1:**
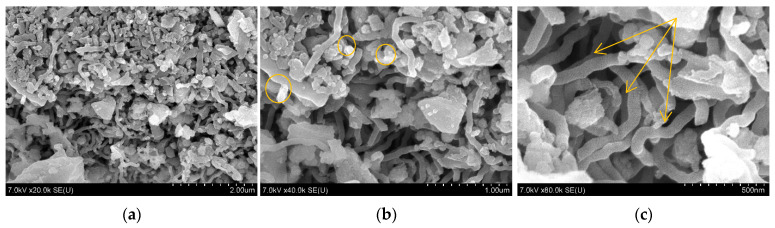
SEM images of the CSF at different magnifications. (**a**) ×20.0 K; (**b**) ×40.0 K; (**c**) ×80.0 K.

The morphology and structure of the CSF sample were analysed by scanning electron microscopy (SEM) using a Hitachi S-4800 scanning electron microscope (Hitachi High-Technologies Corporation, Tokyo, Japan) at various magnifications (Figure 1).

The SEM micrographs show that the CSF structure resembles a mesh of interwoven fibres (Figure 1c), which is attributed to the plant-based origin of the material. The images also reveal the presence of amorphous silica inclusions (Figure 1b) and inorganic compounds in the form of agglomerates composed of layered structures.

**Figure 2:**
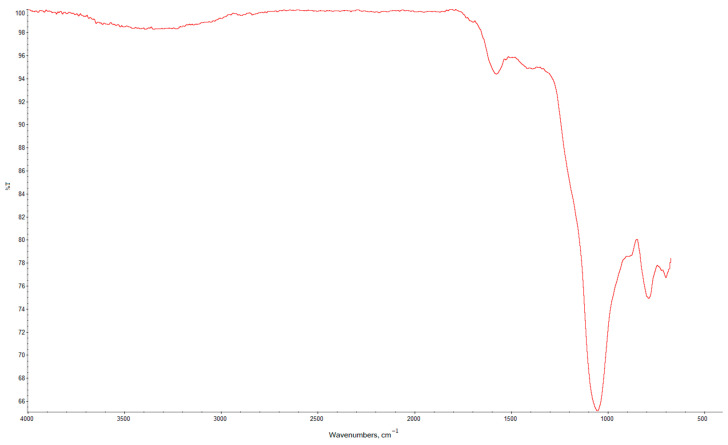
FTIR spectrum of the CSF sample.

An infrared spectrum of the CSF sample was obtained using Fourier-transform infrared (FTIR) spectroscopy with a Nicolet iN10 infrared microscope (Thermo Scientific, Madison, WI, USA) (Figure 2).

FTIR analysis performed using a NEXUS E.S.P. Fourier-transform spectrometer for the studied CSF revealed the following characteristic absorption bands:

- 1730 cm^−1^ (stretching vibrations of C=O groups) [46];
- 1700 cm^−1^ (bending vibrations of hydrogen-bonded OH groups) [45];
- 1595 cm^−1^ (stretching vibrations of COO^−^ groups);
- 1418 cm^−1^ (bending vibrations of Si–C groups);
- 1051 cm^−1^ (stretching vibrations of siloxane groups –Si–O–Si–) [47];
- 790 cm^−1^ (bending vibrations of siloxane groups –Si–O–Si–) [46];
- 699 cm^−1^ (bending vibrations of Si–C groups) [48].

Given the presence of silanol groups on the surface of the carbon–silicon filler and its high content of amorphous silica, a partial replacement of the commercial silica filler with CSF was deemed of interest. The aim was to evaluate the effect of this substitution on the key properties of tread rubber compounds. Partly replacing the highly active silica filler with CSF was performed in the industrial formulation used for the tread of summer car tyres (Table 5).

### 2.2. Methods

#### 2.2.1. Preparation of Rubber Compounds

**Figure 3:**
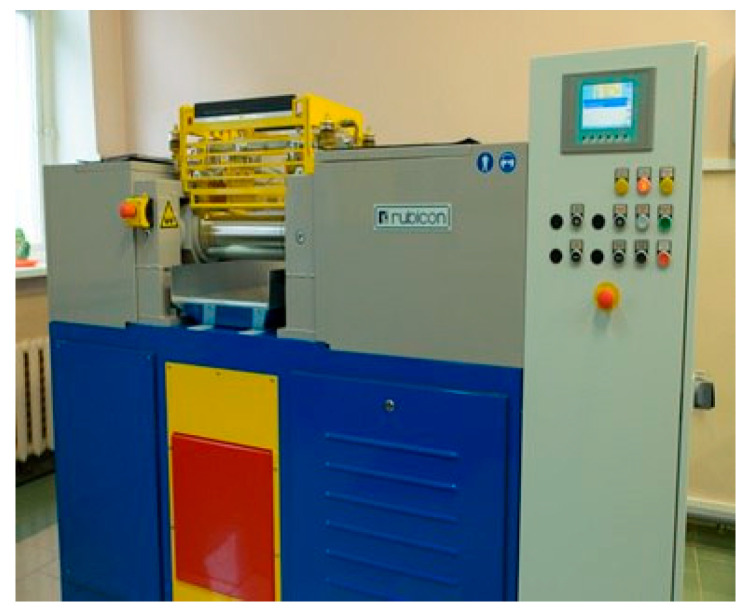
Laboratory two-roll mill RC-WW 330 150/150.

The elastomeric compounds were prepared using a three-stage mixing process on laboratory two-roll mills RC-WW 330 150/150 (Rubicon, Halle, Germany) (Figure 3). At the first stage, styrene–butadiene rubber was mixed with silica/CSF, silane, dispersant, plasticiser, zinc salt of fatty acids, resin, zinc oxide, and masterbatch. The discharge temperature from the mixer was 145–150 °C.

The base compound obtained from the first stage was mixed with antioxidants at the second stage. The discharge temperature from the mixer was also maintained at 145–150 °C.

At the third stage, the vulcanising system was introduced into the compound obtained from the second stage. The discharge temperature at this stage was 95–100 °C.

#### 2.2.2. Determination of Plastoelastic Properties of Rubber Compounds

The plastoelastic properties of rubber compounds describe their behaviour during the shaping of workpieces before vulcanisation. These properties influence both the efficiency of the technological process and the quality of the final product.

**Figure 4:**
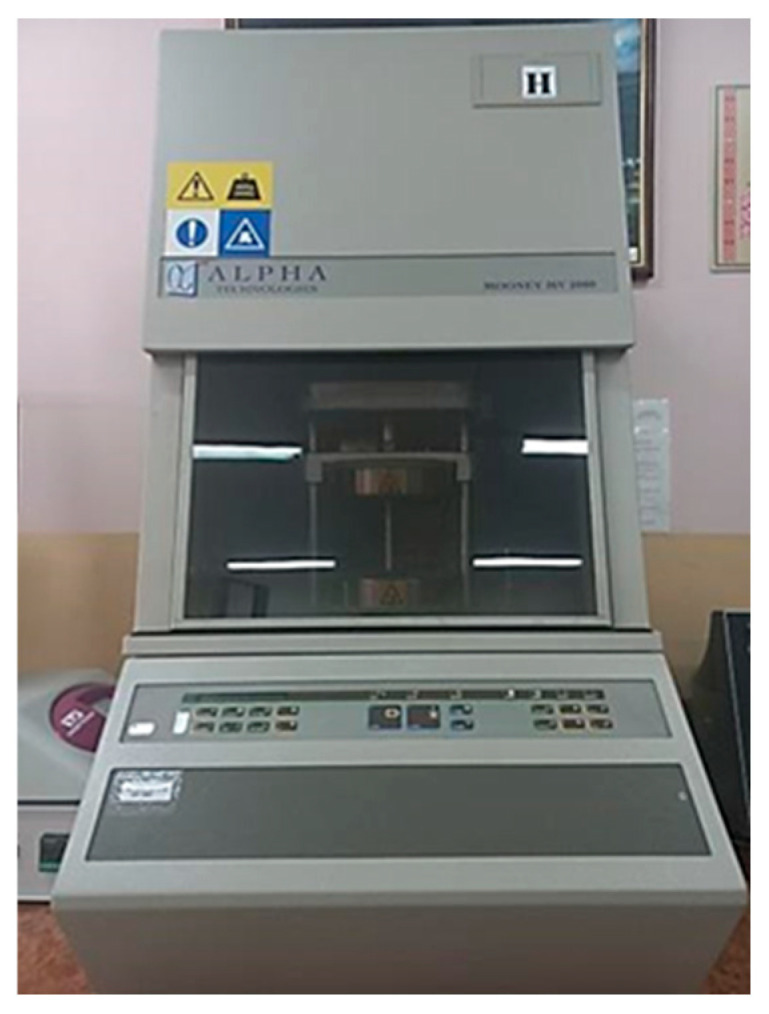
Mooney viscometer MV2000.

Mooney viscosity was determined using a disc-type rotational viscometer MV 2000 (Alpha Technologies, Hudson, OH, USA) according to standard methodology (Figure 4) [49]. In this method, the polymer sample is subjected to circular shear at a constant rate in a thin annular layer. The torque generated in the sample is proportional to the polymer’s viscosity.

#### 2.2.3. Determination of Plasticity and Elastic Recovery of Rubbers

**Figure 5:**
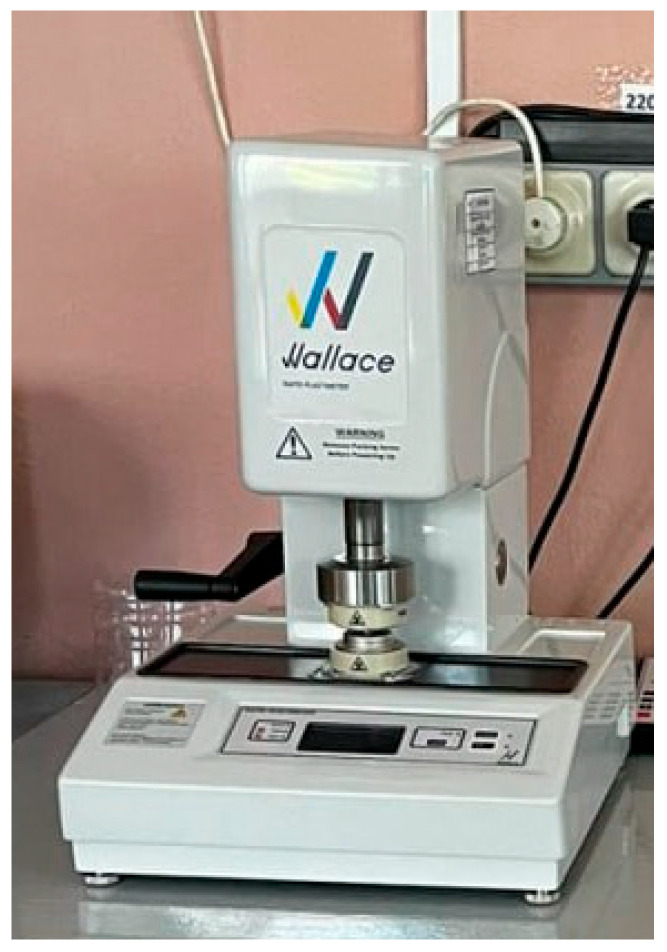
Plastometer model P.12E.

According to standard methodology [50], the plasticity (P) of the rubber compounds was determined using a Wallace plastometer, model P.12E (Wallace, Surrey, UK) (Figure 5), with a rigid system mass of 5.00 ± 0.01 kg and a testing temperature of 70 ± 1 °C. This parameter is defined as the ratio of residual deformation to the average sample height under load and is calculated using Equation (1).

Elastic recovery (R′) was calculated using Equation (2) as the difference between the sample heights after removal of the load (and resting for 3 min outside the device at 23 ± 2 °C) and under load in the plastometer (at 70 ± 1 °C).

(1) $$P=S\cdot R=\frac{h_{0}-h_{2}}{h_{0}+h_{1}}$$

(2) $$R^{\prime }=\frac{h_{2}-h_{1}}{h_{0}-h_{1}}$$

where

*h*_0_—initial height of the specimen;

*h*_1_—height of the specimen under load;

*h*_2_—height of the specimen after load removal and relaxation, mm.

Plasticity and elastic recovery are expressed in relative units ranging from 0 to 1.

#### 2.2.4. Determination of Vulcanisation Kinetic Parameters of Rubber Compounds

The vulcanisation kinetics of the rubber compounds were determined by measuring the torque under shear deformation caused by oscillations of a biconical rotor at a specified frequency and amplitude under controlled sample temperature conditions [51].

**Figure 6:**
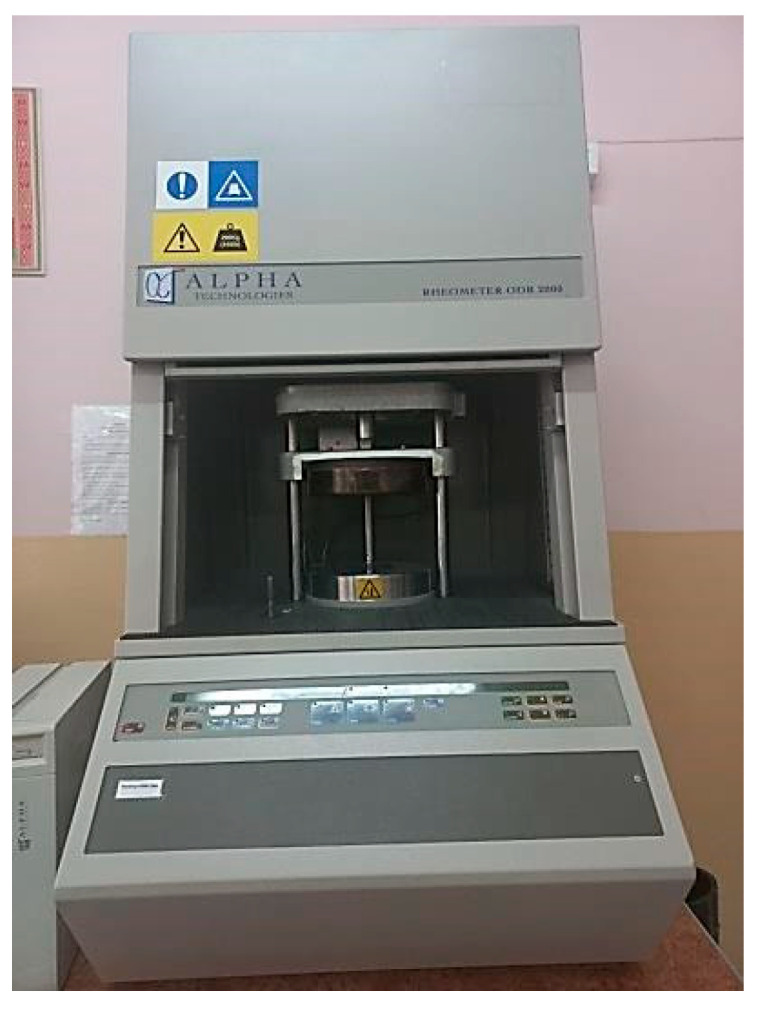
Rheometer ODR 2000.

The tests were performed using an ODR 2000 rheometer (Alpha Technologies, Hudson, OH, USA) (Figure 6). Based on the resulting rheological curves, the following parameters characterising the rheological and vulcanisation behaviour of the compounds were determined:

M_L_ represents the minimum torque, corresponding to the lowest value on the vulcanisation curve, proportional to the viscoelastic properties of the rubber compound at vulcanisation temperature and indicative of compound stiffness, dN·m;

M_H_ represents the maximum torque, corresponding to the peak value on the vulcanisation curve, proportional to the shear modulus of the vulcanised rubber, indicating its final stiffness, dN·m.

ts_2_ represents the scorch time, defined as the time at which torque increases by 2 dN·m above ML, min.

tc(90) represents the optimum vulcanisation time, at which the compound reaches optimal curing properties; it may differ from the actual time required to achieve maximum performance, min.

ΔM represents the torque difference between M_H_ and M_L_, dN·m.

Vulcanisation of the SBR-based rubber compounds was conducted at 143 °C for 60 min.

#### 2.2.5. Methods for Determining Physico-Mechanical Properties of Vulcanisates and Rubber Compounds

**Figure 7:**
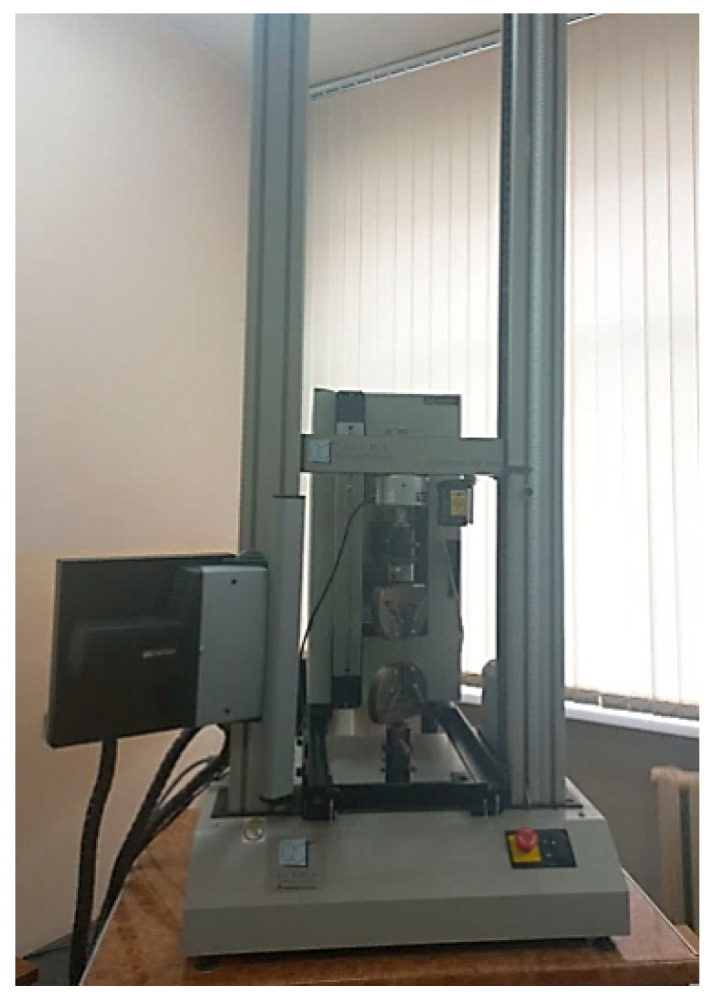
Tensile testing machine T2020 DC10 SH.

The elastic and strength characteristics of the elastomeric compositions were assessed based on tensile strength, stress at a specified elongation, and elongation at break, using a T2020 DC10 SH tensile testing machine (Alpha Technologies, Hudson, OH, USA) (Figure 7) according to standard methodology [52].

Ageing resistance was evaluated by measuring changes in these properties after ageing in a thermostat at 120 ± 2 °C for 12 ± 0.25 h. Tests were performed using standard procedures [53] using a sectional thermostat model TVS-1. Ten standard specimens were tested per rubber formulation.

**Figure 8:**
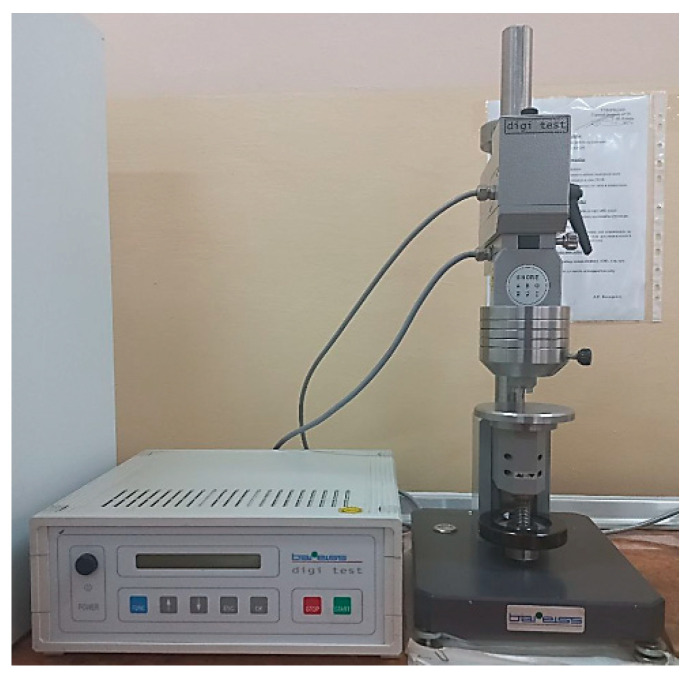
Hardness tester DIGI-TEST.

Shore A hardness of the elastomeric compositions was determined before and after thermal ageing (100 ± 2 °C for 12 ± 0.25 h) using a DIGI-TEST durometer (Bareiss, Oberdischingen Germany) (Figure 8) on three specimens, following standard methodology [54].

**Figure 9:**
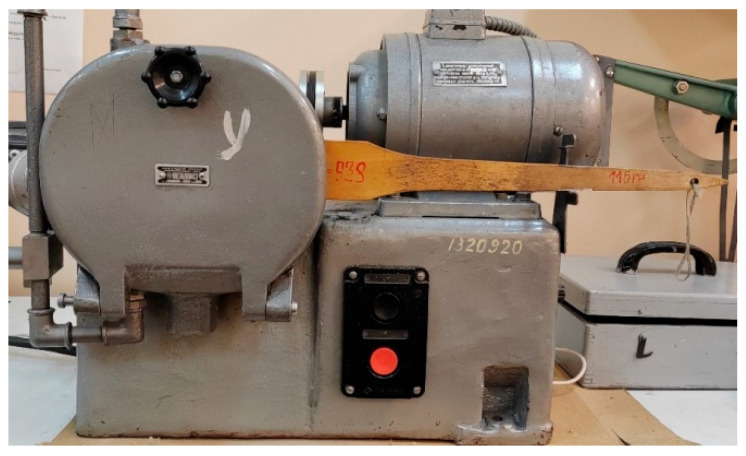
Testing machine MI-2.

Following standard procedure, abrasion resistance under sliding on a renewable surface, simulating tread wear, was assessed using the MI-2 abrasion tester (Figure 9) [55]. The normal applied force on two specimens was 26 N (2.6 kgf). Depending on the wear resistance of the rubber, the test duration was adjusted, ensuring that mass loss did not fall below 0.05 g.

#### 2.2.6. Determination of Rubber Compound Tackiness

**Figure 10:**
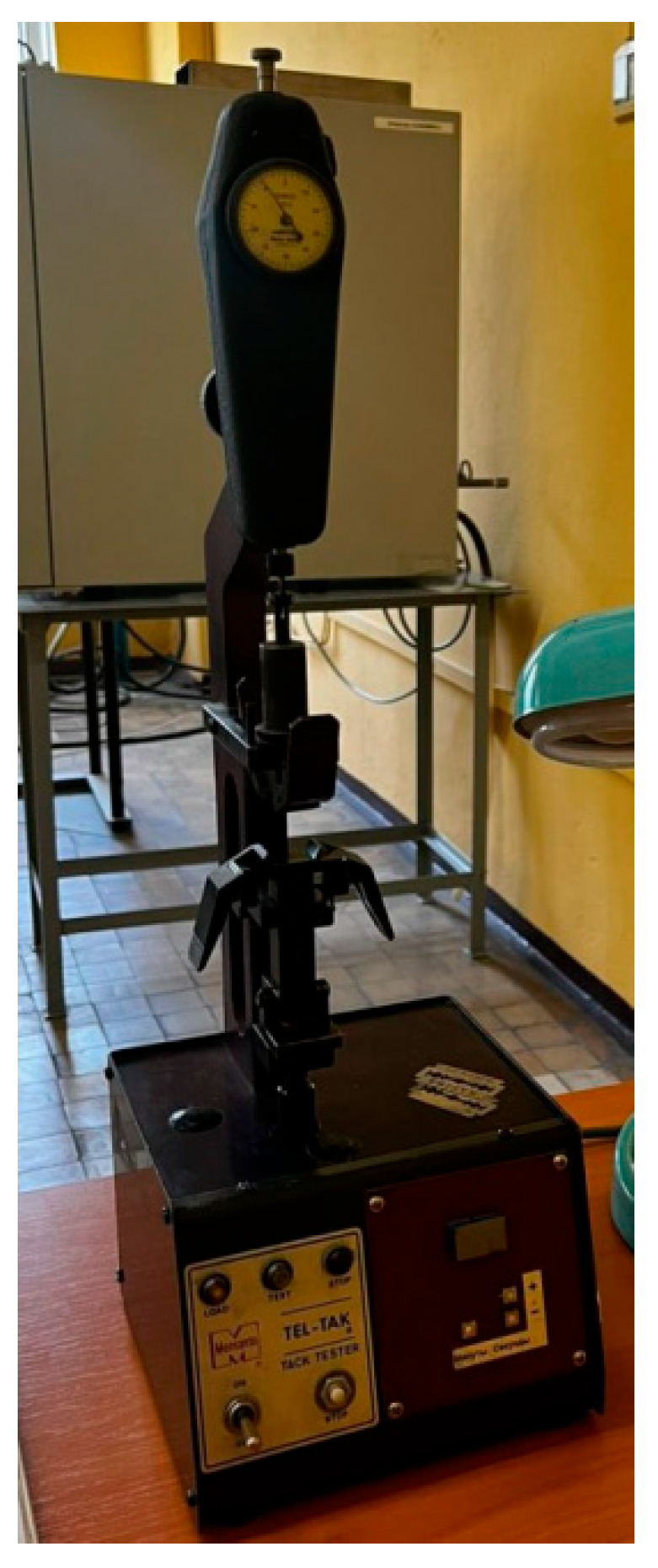
Tack tester Tel-Tak.

Tackiness of the rubber compounds was assessed by measuring the conditional stress required to separate two identical rubber specimens after a 30 s contact under a 16-ounce load at a deformation speed of 25.44 mm/min. Testing was conducted using a Tel-Tak device (Monsanto, St. Louis, MI, USA) (Figure 10).

#### 2.2.7. Methods for Determining Viscoelastic Properties of Tread Rubbers

**Figure 11:**
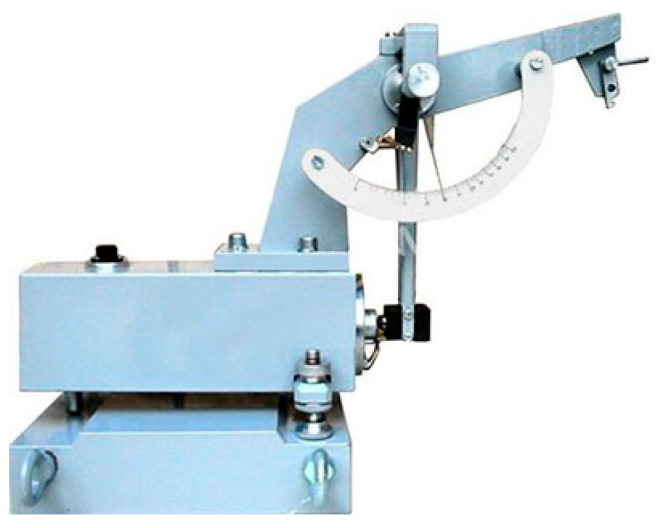
Schob-type resilience tester.

The rebound elasticity of the elastomeric compounds was determined according to the standard method [56] using a Schob-type resilience tester (Figure 11).

The method involves measuring the rebound height of a pendulum hammer dropped from a specified height onto the sample. The test samples were disk-shaped, with parallel surfaces, a minimum diameter of 29 mm, and a 6.00 ± 0.25 mm thickness. Sample surfaces must be smooth, bubble-free, and free from defects or contaminants. Curing time and pre-test conditioning of samples were performed according to standard methodology [57]. Tests were conducted at 23 ± 2 °C and 100 ± 1 °C on no fewer than two specimens.

**Figure 12:**
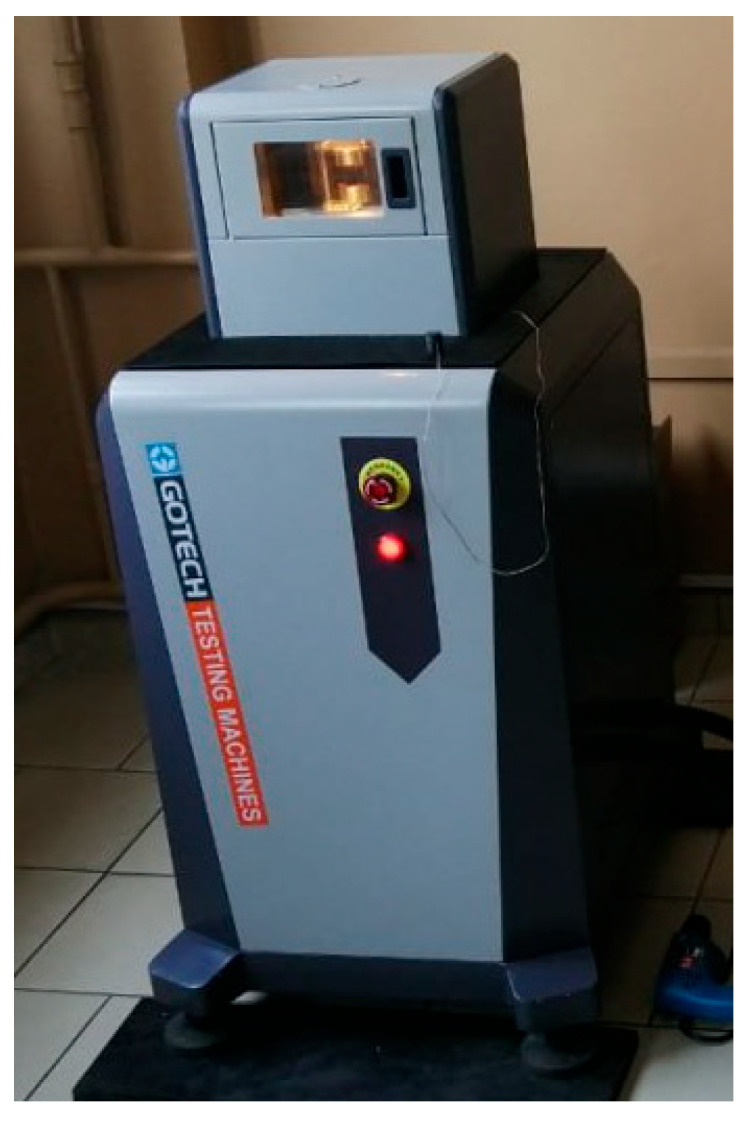
Flexometer model RHU-3000N.

Heat build-up was evaluated following standard procedure [58] using a Goodrich-type flexometer (RHU-3000N, Gotech Testing Machines, Dongguan Co., Ltd., Dongguan, China) (Figure 12). The test involved repeated specimen compression under specified conditions (30 Hz frequency, 4.45 ± 0.03 mm stroke, 1.00 ± 0.03 MPa stress) for 25 min (±5 s), followed by measurement of sample temperature and residual deformation after a rest period.

**Figure 13:**
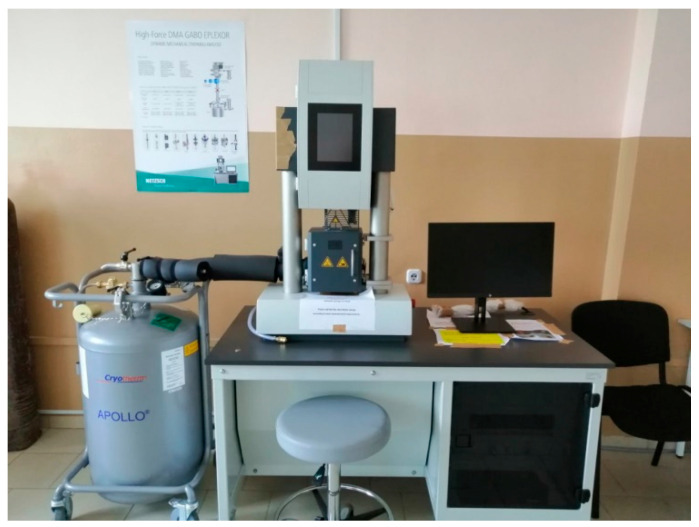
DMA GABO Eplexor 500N.

For the quantitative assessment of the viscoelastic properties of rubber under harmonic dynamic loading, the storage modulus (E′) and loss modulus (E″) are commonly used. Their determination was carried out using a dynamic mechanical analyser, DMA GABO Eplexor 500N (Netzsch, Selb, Bavaria, Germany) (Figure 13). Phase shift between stress and strain leads to dynamic hysteresis, causing mechanical energy losses and heat build-up. These losses are proportional to the phase angle [59].

The loss tangent (tan δ), defined as the ratio of loss modulus to storage modulus (E″/E′), characterises the extent to which the material exhibits viscous versus elastic behaviour. A higher tan δ value indicates more pronounced viscous behaviour [60].

Tests to determine viscoelastic losses in tread rubbers were conducted according to ISO 6721 [61] using a dynamic mechanical analyser, DMA GABO Eplexor 500N (Netzsch, Selb, Bavaria, Germany). Samples were subjected to cyclic compression at a static load of 0.56 MPa, a dynamic load of 0.50 MPa, and a constant frequency of 11 Hz. The temperature range was 30 to 80 °C, with a 2 K/min heating rate.

## 3. Results and Discussion

### 3.1. Technological Properties of Rubber Compounds

#### 3.1.1. Determination of Mooney Viscosity of Rubber Compounds

One of the most important methods for assessing the processing properties of rubber compounds is the determination of Mooney viscosity [52]. The viscosity of rubber compounds measures the effort required to make the material flow at a given rate at a particular stage of processing [62]. There are critical viscosity limits beyond which the processing of rubber compounds becomes economically inefficient and technically unfeasible [63].

**Figure 14:**
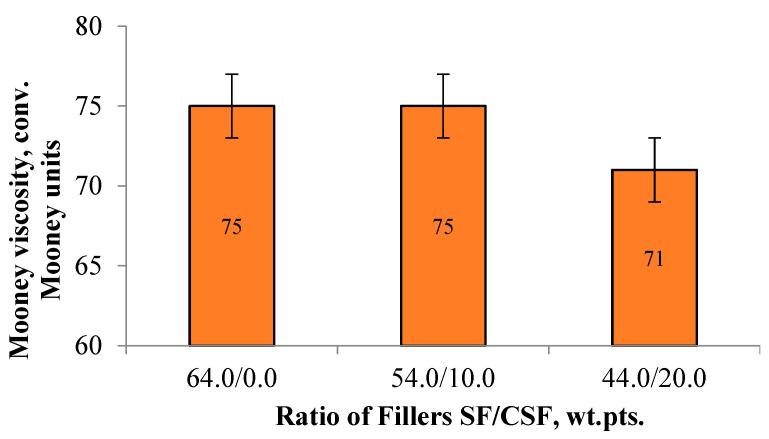
Dependence of the Mooney viscosity of tread rubber compounds on the dosage of CSF.

It was found that replacing the industrial high-reinforcing silica filler with the investigated carbon–silica filler derived from plant-based raw materials at a dosage of 10.0 wt. pts. does not affect the Mooney viscosity of rubber compounds intended for tyre treads. However, increasing the content of the carbon–silica filler to 20.0 wt.pts. leads to a decrease in this parameter by 4 conditional units (5.3%) (Figure 14).

The observed trend in the Mooney viscosity of rubber compounds with increasing content of the CSF is primarily attributed to differences in the agglomerate sizes of the fillers: the average agglomerate size of CSF is up to 12 µm, whereas that of the SF reaches up to 15 µm. This difference significantly reduces the ability of the mixed filler system to form chain-like structures, thereby weakening the physical filler–rubber interactions. As a result, the mobility of elastomeric macromolecular segments increases, shear deformations intensify, and macromolecular displacement becomes more facile, decreasing viscosity [25,29,55]. Furthermore, the mismatch in surface energy between the two fillers prevents the formation of a unified filler network. Studies [25,64] have demonstrated a reduction in Mooney viscosity of rubber compounds when rice husk ash is used in tyre rubber formulations, compared to compounds containing either carbon black or the silica filler Zeosil 1165 MP. This decrease in viscosity is attributed to the lower specific surface area of rice husk ash, which in turn leads to reduced bound rubber content and a diminished in-rubber structure effect on the elastic modulus.

#### 3.1.2. Plasticity and Elastic Recovery of Rubber Compounds

While viscosity is defined as resistance to plastic deformation (P), the term “plasticity” refers to the “ease of deformation” of a rubber compound sample. In some respects, “plasticity” and “viscosity” describe the same property but with opposite meanings [25]. Viscosity characterises a system’s resistance to shear stress, whereas plasticity and stiffness reflect its resistance to compressive deformation. Elastic recovery (R′) describes the shrinkage behaviour of compounds and semi-finished products. The evaluation of plasticity and elastic recovery provides insight into the processability of rubber compounds on industrial equipment [65].

Table 6 presents the results of plasticity (P) and elastic recovery (R′) measurements for tread rubber compounds.

Table 6 shows partial replacement of the SF with the CSF at up to 20.0 wt.pts. In SBR-based compounds, this results in a 7.7% increase in plasticity. These results are consistent with the Mooney viscosity data obtained using a shear viscometer.

It was also found that the elastic recovery of the studied compounds decreased by 11.1% compared to the reference sample, indicating reduced shrinkage. This, in turn, may require formulation adjustments and optimisation of calendering process parameters.

#### 3.1.3. Kinetic Parameters of the Vulcanisation Process

Vulcanisation is a complex set of physicochemical processes occurring in rubber compounds. These primarily crosslink rubber macromolecules via chemical bonds of various energies and natures, forming a three-dimensional vulcanisation network. The formation of crosslinks proceeds through a series of chemical interactions involving vulcanising agents and accelerators [63]. The vulcanisation process is influenced by all ingredients present in the rubber compound.

**Figure 15:**
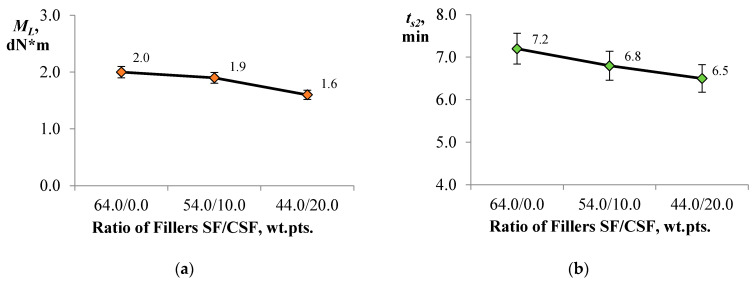

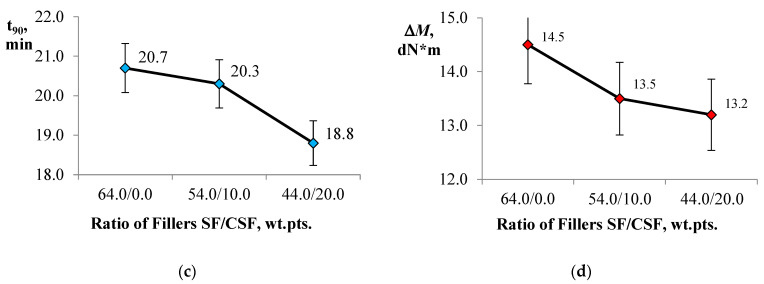
Influence of filler ratio on the kinetic parameters of rubber vulcanisation: (**a**) minimum torque; (**b**) scorch time; (**c**) optimum cure time; (**d**) torque difference between maximum and minimum values.

The results of the study on the kinetic parameters of rubber compound processing—where the carbon–silica filler was used as a partial replacement for the highly reinforcing SF in elastomer formulations intended for the tread portion of tyres—are presented in Figure 15a–d.

As shown in Figure 15, incorporating CSF as a partial replacement for the highly reinforcing SF at up to 20.0 wt.pts. affects the crosslinking process in the rubber compound. Partial substitution of SF with rice husk-derived carbon filler reduces the minimum torque (M_L_) by up to 20.0% compared to the industrial formulation (Figure 15a). The minimum torque reflects compound viscosity, which is generally associated with polymer chain characteristics and filler-related properties such as type, filler–rubber and filler–filler interactions, and forming a filler network. As filler content increases, the development of a filler network intensifies, leading to an increase in M_L_ [66]. However, in the studied systems, higher CSF content results in a progressive decrease in M_L_, indicating disruption of filler–filler interactions.

This behaviour may be attributed to polar groups (COO^−^, OH^−^) on the CSF surface, which can interact with residual silanol groups on the SF surface (remaining after silanisation), thereby exerting a shielding effect [67]. Additionally, the scorch time (t_s2_) and optimum cure time (t_90_) decreased by 9.7% and 9.2%, respectively (Figure 15b,c), likely due to the porous structure of the rice husk ash, which promotes adsorption of vulcanisation system components on its surface, thereby slowing the crosslinking process [39,67].

Moreover, partial substitution of SF with CSF led to a decrease in ΔM (torque difference) by up to 9.0%, indirectly reflecting the degree of macromolecular crosslinking. This reduction can be attributed to the smaller surface area of CSF relative to SF, which may weaken filler–filler interactions. In addition to the diminished filler–filler network, crosslink density may be further reduced due to a dilution effect. The observed differences in the vulcanisation kinetics are influenced by the amount and type of functional groups on the filler surfaces and their participation in the silanisation process [68].

#### 3.1.4. Tackiness of Rubber Compounds (Without Rest)

Tackiness refers to the ability of an unvulcanised rubber compound to adhere to itself or to another compound within a short contact time and under moderate pressure. This property is critical for rubber products such as tyres and conveyor belts, which are assembled by overlaying one calendered or extruded layer onto another. An unvulcanised rubber product must maintain cohesion until it is placed into a mould or press for vulcanisation.

**Figure 16:**
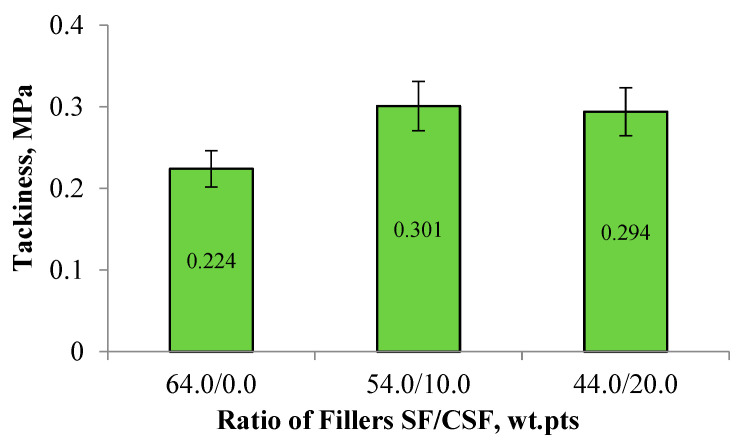
Dependence of rubber compound tackiness on the filler ratio.

Experimental results showed that the CSF under investigation significantly affects the tackiness of the elastomeric compositions. Incorporating 10.0 and 20.0 wt.pts. of CSF into tread compound formulations increases the adhesion strength of duplicated samples by 31.3–34.4% compared to the industrial reference compound (Figure 16).

The increased rubber compound tackiness is likely due to the reduced filler network structure when CSF is used. This structural loosening facilitates the diffusion of rubber macromolecules to the surface of the compound, thereby eliminating phase boundaries and enhancing interfacial adhesion.

### 3.2. Physico-Mechanical Properties of the Compounds Before and After Thermal Ageing

The mechanical strength of rubber is significantly influenced by the chemical composition and configuration of the rubber macromolecules, the type of vulcanising system, the structure formed during vulcanisation, and the concentration and morphology of fillers and other ingredients [69]. The results of tensile and elastic property measurements for SBR-based rubbers containing different SCF loadings are presented in Table 7.

It was found that rubber compounds containing CSF as a partial replacement for the highly reinforcing SF exhibit lower tensile strength both before and after exposure to elevated temperature and atmospheric oxygen. The tensile strength of the SF-based compound before thermal ageing reached 16.4 MPa, while formulations containing CSF ranged from 14.3 to 12.7 MPa. At the same time, the incorporation of CSF had virtually no effect on the elongation at break of the tread rubber compounds.

The observed reduction in mechanical strength in CSF-containing compounds is likely attributable not only to the plasticising effect but also to the lower dispersibility and surface activity of CSF compared to SF. Moreover, the decrease in tensile properties with increasing CSF loading may be associated with the filler’s limited ability to withstand stress transfer from the polymer matrix [70].

**Figure 17:**
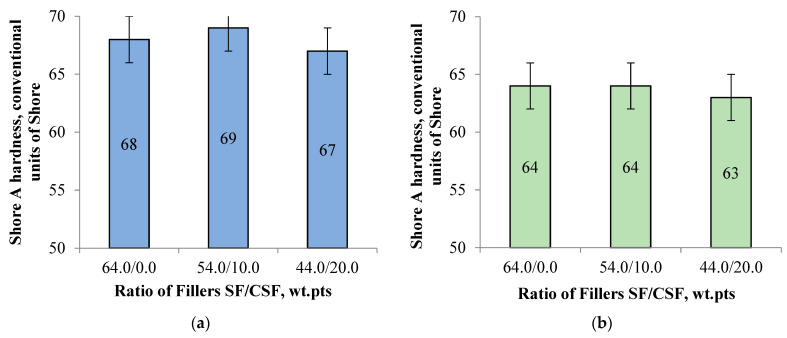
The Shore A hardness variation of the investigated rubbers depending on the filler ratio: (**a**) before thermal ageing; (**b**) after thermal ageing at 100 °C.

It was also established that using CSF as a partial replacement for SF in elastomeric formulations designed for summer tyre treads has no significant effect on Shore hardness. As shown in Figure 17, the variation in Shore hardness does not exceed two units, both before and after thermal ageing at 100 °C.

The wear resistance of tread rubbers is a critical parameter reflecting the operational performance of tyres.

Table 8 summarises the abrasion resistance values of the studied rubber compounds before and after exposure to elevated temperature (120 °C) and atmospheric oxygen.

It was found that incorporating the carbon–silicon filler into tread rubber formulations increases the abrasion rate of the rubber compounds by up to 43.3% before thermal ageing and 22.5% after thermal ageing, respectively. This decrease in abrasion resistance is likely attributed to the reduced content of the highly reinforcing silica filler, which possesses a high specific surface area. Its reduction results in fewer chemical interactions between the rubber matrix and silanol groups, consequently decreasing the mechanical strength of the rubber (Table 7) and promoting higher material wear [52]. Additionally, the coarse dispersion of carbon–silicon filler particles may contribute to forming voids that act as stress concentrators, reducing mechanical strength and creating surface irregularities that facilitate material detachment during abrasion.

#### Elastic Hysteresis Properties of Tread Rubbers

Since during tyre operation the rubber is subjected to alternating deformations, the amplitude of which is significantly lower than the elongation at break, it is not sufficient to evaluate the product behaviour under such conditions based solely on strength and fatigue characteristics.

The selection of rubbers with optimal properties for the specific features of the dynamic regime during the operation of a particular product should be based on a set of parameters that characterise the relationship between stress and strain under cyclic loading (elastic hysteresis properties) and the correlation between dynamic stress and fatigue life of rubber, i.e., the number of load cycles that a sample of a given material can withstand without failure [60].

Relatively short-term tests for determining elastic hysteresis properties include rebound resilience testing, heat build-up measurement, fatigue resistance, and determination of hysteresis losses [52].

The elastic modulus E′ characterises the portion of mechanical work that is stored as elastic deformation and is reversibly released during unloading, i.e., the storage modulus E′ defines the material’s elasticity and its ability to retain energy. The elastic modulus of rubber significantly influences tyre–road grip, which depends on the type and condition of the road surface, the tyre’s construction and purpose, driving speed, and the frictional properties of the tread rubbers, among other factors [59,71].

**Figure 18:**
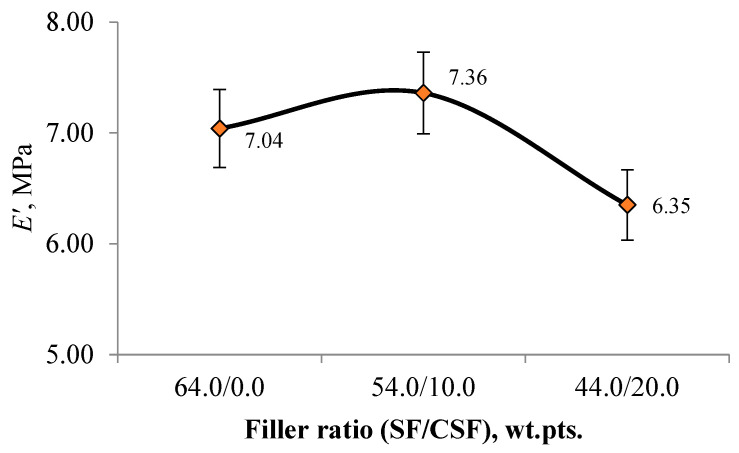
Dependence of elastic modulus on the filler ratio.

The dependence of the elastic modulus E′ at 60 °C of the studied rubbers on the filler ratio is shown in Figure 18.

Based on the obtained data, replacing 10 wt.pts. of SF with CSF resulted in a 4.5% increase in the storage modulus of the rubber compounds, potentially improving the resistance of the tread to mechanical damage. However, when the CSF content was increased to 20 wt.pts, the storage modulus decreased by 9.8%, indicating increased material elasticity, which may positively affect tyre–road adhesion.

The loss modulus (E″) represents the portion of mechanical energy irreversibly dissipated as heat during each deformation cycle. A higher E″ value indicates increased heat generation during operation, which results in greater thermal energy loss to the environment. This leads to increased fuel consumption and negatively affects the wear resistance of the rubber compounds.

**Figure 19:**
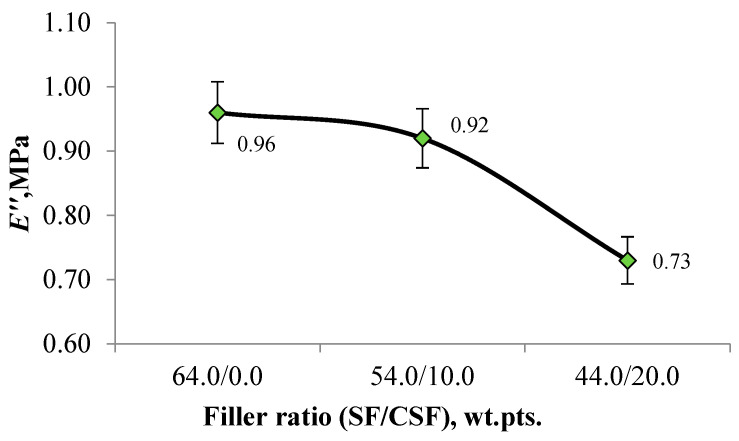
Dependence of loss modulus on the filler ratio.

The results of the loss modulus (E″) measurements for the investigated vulcanisates are presented in Figure 19.

Using carbon–silicon filler in tread rubber compositions as a partial replacement for highly reinforcing SF has a positive effect on the loss modulus E″. In particular, SF was partially replaced with the investigated CSF at dosages of 10.0 and 20.0 wt.pts. leads to a reduction in the loss modulus by 4.2% and 24.0%, respectively. Therefore, using CSF in the studied elastomeric composition reduces heat loss to the environment, which can lower fuel consumption and improve the wear resistance of the rubber during real-world operation [63,70].

**Figure 20:**
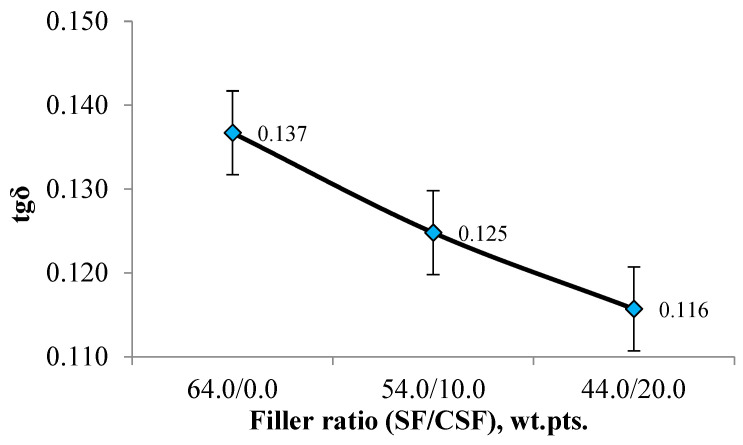
Dependence of the mechanical loss tangent on the filler ratio.

To evaluate the performance characteristics of tyre tread rubber in laboratory conditions, the values of the mechanical loss tangent (tgδ) at 60 °C are used. These values are obtained from dynamic mechanical analysis, which reflects the energy loss due to hysteresis during dynamic deformations and is associated with tyre rolling resistance [72,73]. Figure 20 shows the dependence of tgδ on the filler ratio in the studied elastomeric compositions.

Based on the obtained data, an increase in the dosage of CSF leads to a decrease in the mechanical loss tangent by 7.1–14.3% compared to the composition filled only with highly reinforcing SF. This indicates that elastomeric compositions containing carbon–silicon filler exhibit lower hysteresis losses. It can be assumed that the reduction in the elastic modulus, loss modulus, and mechanical loss tangent in rubbers containing CSF is due to the lower specific surface area of the natural filler compared to the highly reinforcing silica filler. At the same time, the industrially used SF, which has a higher specific surface area, adsorbs a larger amount of polymer on its surface, resulting in the formation of a dense interfacial layer and enhanced adhesion between the polymer and the filler particles [74,75,76].

Table 9 presents the results of the study on the effect of CSF dosage on the viscoelastic properties of tread rubber elastomeric compositions.

As seen from the data in Table 9, the introduction of CSF into tread rubber compounds at all investigated dosages leads to a slight increase in rebound resilience under both normal conditions (23 ± 2 °C) and elevated temperatures (100 ± 1 °C). This effect can be attributed to the lower specific surface area of CSF microparticles compared to that of the highly reinforcing SF, which results in reduced filler–filler interactions, lower energy losses, and decreased hysteresis, thereby enhancing the elasticity of CSF-containing rubbers.

The results of the Goodrich heat build-up test demonstrated partial replacement of SF with CSF in dosages of 10.0 and 20.0 wt.pts. contributes to a decrease in the internal sample temperature by 2 °C and 7 °C, respectively. In addition, substituting the industrial filler with CSF reduced the overall heat build-up by up to 8 °C. This observed trend can be explained by reduced internal friction due to the lower crosslink density of macromolecular chains.

It is also noteworthy that, according to the data in Table 9, tread rubbers containing CSF exhibit improved fatigue resistance (22.5 and 24.3 thousand cycles) compared to the industrial blend (21.6 thousand cycles). This improvement may be attributed to the presence of numerous hydrogen bonds between surface silanol groups on the SF particles, which are thermally unstable and tend to break at elevated temperatures. This increases internal friction losses, reduces elasticity, and enhances heat generation.

The results of this study on the effect of partial replacement of a commercial highly reinforcing silica filler (white carbon black grade Extrasil 150VD) with a carbon–silicon filler derived from rice production waste (CSF) in industrial elastomeric compounds based on styrene–butadiene rubber (SBR) show good agreement with the general mechanism of filler reinforcement developed in the elastomer reinforcement theory by Grishin B.S. et al. and summarised in the monograph “Theory and Practice of Elastomer Reinforcement” [70].

According to this theory, the structure of reinforcing fillers such as carbon black and silica varies depending on the filler grade, which differs in the size of the primary particles (the smallest dispersed unit formed by the fusion of individual particles) and the morphology of aggregates. Depending on the size of the primary structure, fillers may exhibit high or low degrees of aggregation. The primary aggregates of reinforcing fillers are capable of forming chain-like or clustered secondary structures (agglomerates), which are disrupted into primary aggregates and smaller agglomerates during rubber mixing. The strength of these agglomerates increases with decreasing size of primary particles and aggregates, and with a lower structural index. Typically, aggregates continue to form larger agglomerates held together by physical forces. Highly reinforcing fillers with high dispersity usually contain a large proportion of branched aggregates, thus exhibiting high structurality.

Furthermore, branched aggregates form porous structures with large surface areas available for interaction with the polymer matrix, leading to improved reinforcement. Reinforcing fillers enhance the strength of rubber and improve other properties, such as tear and abrasion resistance and stress at a given strain. As filler structurality decreases, a greater proportion of spheroidal and ellipsoidal aggregates appears. Fillers with low structurality lack pronounced reinforcing effects but are used industrially to reduce the cost of rubber compounds. At the same time, the processability of the compounds improves due to the hydrodynamic effect (reduction in Mooney viscosity, improved processing kinetics, and enhanced dispersion quality), and the rubber materials acquire specific properties such as heat and light resistance.

In this work, a carbon–silicon filler was used, which is an inert material of plant origin and is characterised by a low specific external surface area (Table 3) and thus by lower structurality compared to the highly reinforcing silica filler (Extrasil 150VD). The results showed a reduction in Mooney viscosity of the rubber compounds containing the carbon–silicon filler, attributed to the difference in aggregate size, which significantly limits the ability to form chain-like filler structures and, consequently, filler–rubber physical interactions. As a result, the mobility of elastomeric macromolecular segments increases, shear deformations become more pronounced, and molecular movement becomes easier, leading to reduced viscosity. At the same time, rubber plasticity increases and the minimum torque decreases, consistent with the viscosity data.

Changes in scorch safety and optimum cure time are attributed to the porous structure of rice husk ash, which promotes adsorption of vulcanisation system components on its surface, thereby slowing down the curing process. The reduction in mechanical strength is not only due to the low dispersity and lack of surface functional groups capable of forming chemical bonds but also to the filler’s inability to bear stresses transferred from the polymer matrix. Improvements in viscoelastic performance and reduced heat build-up are linked to the formation of a looser crosslinked network in the presence of the carbon–silicon filler. A looser network implies greater distances between rubber macromolecules and fewer chain-like filler structures, resulting in increased elasticity and reduced internal heat generation during product use.

## 4. Conclusions

The use of rice husk and stem ash (carbonisate, referred to as CSF) in styrene–butadiene rubber (SBR)-based compounds as a partial replacement (up to 20 phr) for the commercial highly reinforcing silica filler (HSF, Extrasil 150VD) led to a reduction in Mooney viscosity by up to 5.3% and in optimum vulcanisation time by up to 9.2%, thereby lowering the energy consumption of the processing stage. It was found that the incorporation of CSF at all investigated dosages increased resistance to plastic deformation by up to 7.7% and improved tackiness of the rubber compounds by 31.3–34.4%, which is beneficial for enhancing the bonding strength between tyre layers during assembly.

Moreover, partial replacement of HSF with CSF resulted in a decrease in the loss modulus by up to 24.0% and in the mechanical loss tangent by up to 14.3%, indicating reduced heat generation within the tyre and improved fuel efficiency. Performance tests of tread rubber compounds demonstrated an increase in fatigue life by up to 2.7 thousand cycles and a decrease in internal sample temperature by up to 7 °C when using CSF, which is attributed to reduced filler–filler interactions, lower internal friction losses, and diminished hysteresis.

However, a moderate decrease in mechanical strength was observed: the tensile strength, both before and after thermal ageing, decreased by 10.7–27.0%, and abrasion resistance declined by up to 43.3%, both under standard and thermally aged conditions.

The results of this study indicate that rice husk ash is a viable alternative to conventional silica fillers used in the tyre industry. The properties of elastomeric compounds filled with rice husk ash can be further enhanced by reducing particle size and applying physical and/or chemical surface modifications (e.g., wet milling, gamma irradiation, or electron beam treatment), as well as by optimising the rubber compound formulation, particularly the vulcanising system. Such materials can be effectively employed in tyres, fulfilling requirements for environmental sustainability and safety, while also improving processing efficiency and energy savings.

## Figures and Tables

**Table 1:** Basic formulation of the rubber compound for the tread part of a summer tTyre.

Ingredient	Content, wt.pts.
SBR (Manufacturer: Sibur Company, Sterlitamak, Russia)	60.0
Carbon black masterbatch (Manufacturer: JSC “Nizhnekamsktekhuglerod”, Nizhnekamsk, Russia)	50.0
Plasticiser (Manufacturer: Lukoil, Russia, Volgograd)	9.0
Vulcanising system (Manufacturer: Zhengzhou Double Vigour Chemical Product Co., Ltd., Zhengzhou, China)	7.3
White carbon black grade, Extrasil 150VD (Manufacturer: LLC “Kometa”, Tula, Russia)	64.0
Zinc salt of fatty acid (Manufacturer: Chelyabinsk Chemical Plant Oxide, Chelyabinsk, Russia)	5.5
Carbon black N339 (Manufacturer: JSC “Nizhnekamsktekhuglerod”, Nizhnekamsk, Russia)	50.0
Resin (Manufacturer: Lukoil, Volgograd, Russia)	0.2
Silane (Manufacturer: Ecopower, Guangzhou, China)	10.4
Antioxidants (Manufacturer: Zhengzhou Double Vigour Chemical Product Co., Ltd., Zhengzhou, China)	6.0

**Table 2:** Characteristics of the silica filler.

Parameter	Value
Mass fraction of silicon dioxide (SiO_2_), %	97.0
Moisture content, %, not more than	4.0–7.0
pH of aqueous suspension	5.4–7.5
Mass fraction of water-soluble substances, %, max	2.5
Appearance	White microgranules
Specific surface area (nitrogen adsorption), m^2^/g	150–175
Specific surface area of cetyltrimethylammonium bromide (CTAB) adsorption, m^2^/g	140–165
Average particle size, nm	5–15
Loss on ignition (at 950 °C), %, not more than	7.0
Specific electrical conductivity (4 g/100 cm^3^), μS/cm, max	1300

**Table 3:** Physicochemical characteristics of the CSF.

Parameter	Value
Specific surface area (nitrogen adsorption), m^2^/g	36
Average aggregate size, µm	9–12
Dibutyl phthalate (DBP) absorption, cm^3^/100 g	45–90
pH of aqueous suspension	5–7
Ash content, %, max	0.45
Carbon content, %, min	45.0
Volatile matter content, %, max	5
Specific electrical resistivity, Ω·m·10^−6^	21,854.4
Mineral impurity content, %, max	2.36
Total phosphorus content, %, max	0.05
Total sulphur content, %, max	0.03
Bulk density, kg/m^3^, min	420
Structure	Amorphous

**Table 4:** Elemental composition of the CSF.

Element	Content, wt%
C	57.89
Si	26.09
O	11.93
K	1.97
Al	0.51
P	0.40
Mg	0.28
Ca	0.34
Cl	0.27
Na	0.10
Fe	0.20
S	0.02

**Table 5:** Filler ratios in the formulations of industrial elastomeric compounds for summer Tyre treads.

SF/CSF Ratio, wt.pts.
64.0/0.0 (industrial reference)
54.0/10.0
44.0/20.0

**Table 6:** Plasticity and elastic recovery of tread rubber compounds.

Filler Ratio SF/CSF, wt.pts.	Plasticity (P)	Elastic Recovery (R′), mm
64.0/0.0 (industrial mix)	0.39 ± 0.02	0.9 ± 0.04
54.0/10.0	0.39 ± 0.02	0.8 ± 0.04
44.0/20.0	0.42 ± 0.02	0.8 ± 0.04

**Table 7:** Elastic and strength properties of the investigated rubber compounds.

Ratio of Fillers SF/CSF, wt.pts	Tensile Strength, MPa		Elongation at Break, %	
	Before ageing	After 12 h of ageing	Before ageing	After 12 h of ageing
64.0/0.0 (industrial mix)	16.4 ± 0.8	15.9 ± 0.8	430 ± 20	390 ± 20
54.0/10.0	14.3 ± 0.7	14.2 ± 0.7	420 ± 20	370 ± 20
44.0/20.0	12.7 ± 0.7	11.6 ± 0.6	420 ± 20	370 ± 20

**Table 8:** Abrasion resistance of tread rubber compounds before and after thermal-oxidative ageing.

Filler Ratio (SF/CSF), wt.pts.	Abrasion Resistance, m^3^/TJ	
	Before ageing	After ageing
64.0/0.0 (industrial blend)	90 ± 4.5	120 ± 6.0
54.0/10.0	114 ± 5.7	133 ± 6.7
44.0/20.0	129 ± 6.5	147 ± 7.4

**Table 9:** Viscoelastic properties of elastomeric compositions for the tyre tread.

Property	Filler Ratio (SF/CSF), wt.pts.		
	64.0/0.0 (Industrial Blend)	54.0/10.0	44.0/20.0
Rebound resilience at 23 ± 2 °C, %	32 ± 1.6	33 ± 1.7	34 ± 1.7
Rebound resilience at 100 ± 1 °C, %	57 ± 2.9	57 ± 2.9	59 ± 3.0
Goodrich heat build-up test:			
–Temperature inside the sample, °C	148 ± 3	146 ± 3	141 ± 3
–Heat build-up, °C	100 ± 3	94 ± 3	92 ± 3
–Fatigue resistance, cycle life, thousand cycles	21.6 ± 1.1	22.5 ± 1.1	24.3 ± 1.2

## Data Availability

The data presented in this study are available on request from the corresponding author due to privacy restrictions.

## Abbreviations

The following abbreviations are used in this manuscript:

SF	silica filler
CSF	carbon–silica filler
SBR	styrene–butadiene rubber
CTAB	cetriltrimethylammonium bromide
RH	rice husks
RS	rice straw
DBP	dibutyl phthalate
SEM	scanning electron microscopy
FTIR	Fourier-transform infrared spectroscopy
